# Supplementary material for: Stabilization of H5 highly pathogenic avian influenza hemagglutinin improves vaccine-elicited neutralizing antibody responses
Source: bioRxiv. 2025 Aug 2:2025.07.30.667762. Preprint. [Version 1] doi: 10.1101/2025.07.30.667762 (PMC12324432; doi:10.1101/2025.07.30.667762)
Supplement: 1 [file NIHPP2025.07.30.667762v1-supplement-1.pdf]

bioRxiv preprint doi: https://doi.org/10.1101/2025.07.30.667762; this version posted August 2, 2025. The copyright holder for this preprint (which was not certified by peer review) is the author/funder, who has granted bioRxiv a license to display the preprint in perpetuity. It is made available under aCC-BY 4.0 International license.

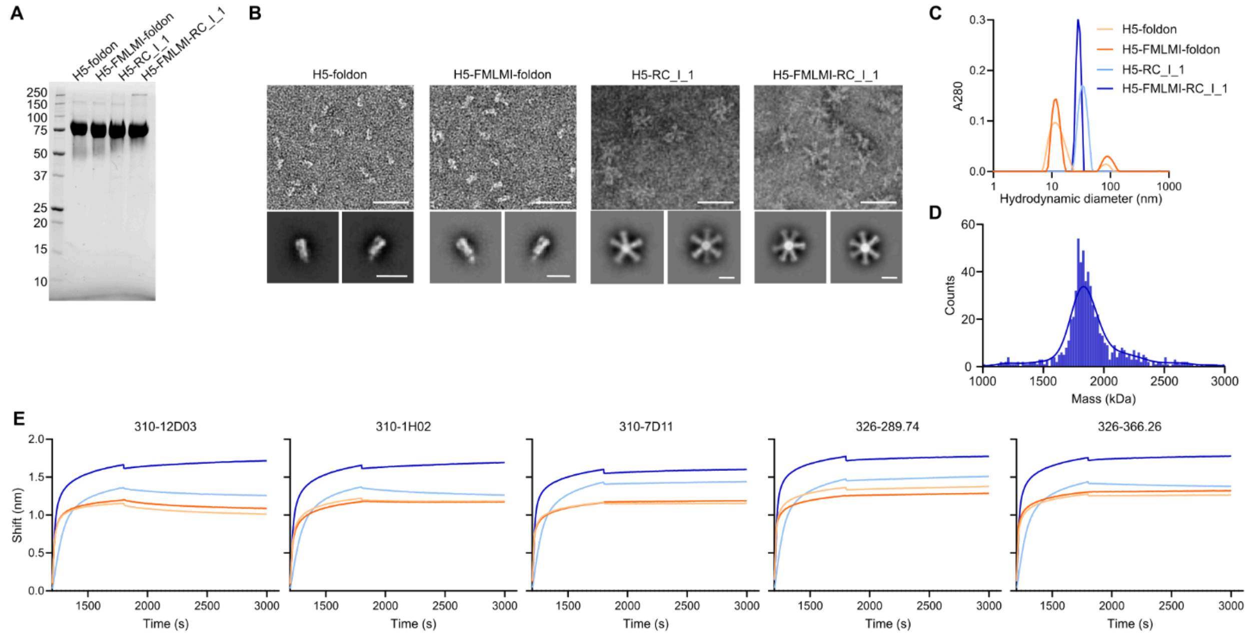

Fig. S1 Biophysical characterization of H5-foldon and nanoparticle constructs.

(A-C) A. Reducing SDS-PAGE, B. nsEM micrographs and 2D averages, and C. DLS of H5 HA constructs. Micrograph scale bars = 50 nm, class average scale bars = 150 nm.

(D) MP histogram and Gaussian fit of H5-FMLMI-RC_I_1.

(E) BLI using anti-RBS and anti-vestigial esterase domain mAbs. Same legend as panel C.

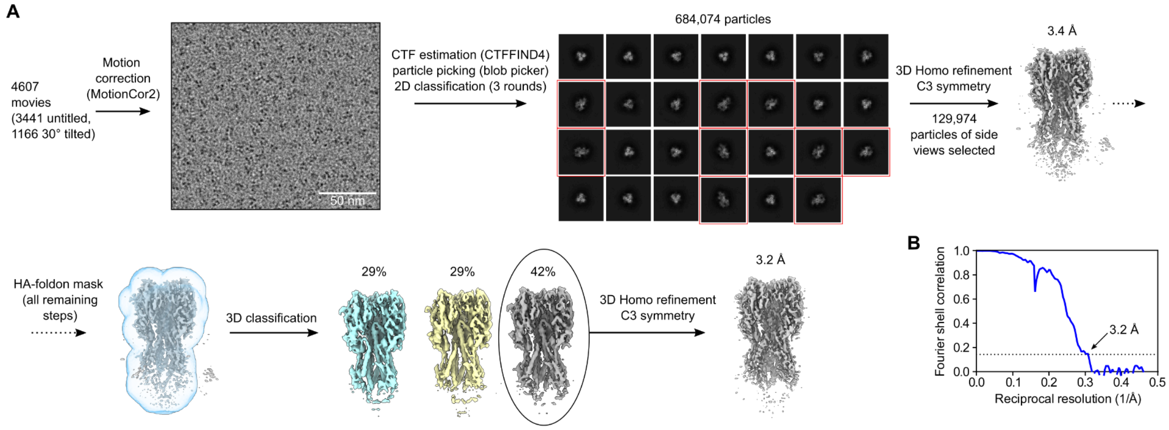

Fig. S2 Cryo-EM processing workflow for H5-FMLMI-foldon.

(A) H5-FMLMI-foldon reconstruction cryo-EM processing diagram. Red boxes around 2D averages show particles of HA-foldon side views selected for further processing.

(B) Gold-standard FSC curve for H5-FMLMI-foldon reconstruction.

bioRxiv preprint doi: https://doi.org/10.1101/2025.07.30.667762; this version posted August 2, 2025. The copyright holder for this preprint (which was not certified by peer review) is the author/funder, who has granted bioRxiv a license to display the preprint in perpetuity. It is made available under aCC-BY 4.0 International license.

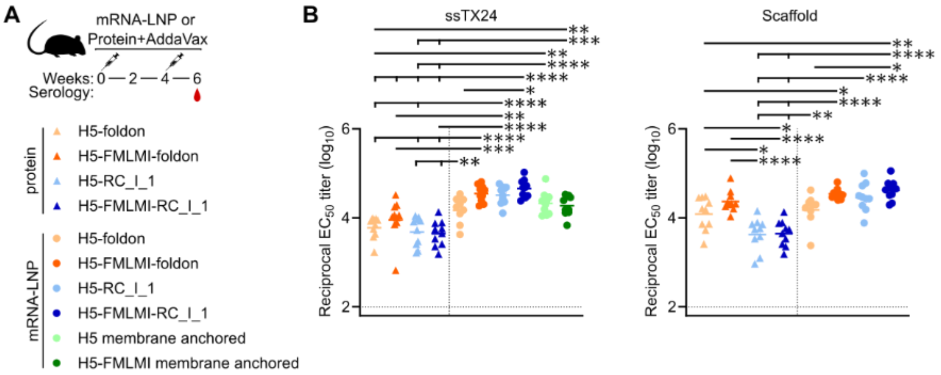

Fig. S3 Vaccine-elicited Antibody Responses in Mice Immunized with H5 protein- and mRNA-LNP-delivered constructs

(A) H5 mouse immunization schedule and groups.

(B) ELISA binding titers in immune sera at week 6 against a stem-only TX24 antigen (left) or RC_I_1 nanoparticle lacking displayed antigen (right).

Each symbol represents an individual animal, and the geometric mean of each group is indicated by the bar (N = 5 rabbits/group). Statistical significance was determined using one-way ANOVA with Tukey's multiple comparisons test; *, p < 0.05; **, p < 0.01; ***, p < 0.001; ****, p < 0.0001.

Table S1. Amino acid sequences of proteins used in this study.

Protein immunogens

>H5-foldon
MENIVLLLAIVSLVKSDQICIGYHANNSTEQVDTIMEKNVTVTHAQDILEKTHNGKLCDLNGVKPLILKDC
SVAGWLLGNPMCDEFIRVPEWSYIVERANPANDLCFPGSLNDYEELKHMLSRINHFEKIQIIPKSSWPN
HETSLGVSAACPYQGAPSFFRNVVWLIKKNDAYPTIKISYNNTNREDLLILWGIHHSNNAEEQTNLYKNPI
TYISVGTSTLNQRLAPKIATRSQVNGQRGRMDFFWTILKPDDAIHFESNGNFIAPEYAYKIVKKGDSTIMK
SGVEYGHCNTKCQTPVGAINSSMPFHNIHPLTIGECPKYVKSNKLVLATGLRNSPLRESRGLFGAIAGFI
EGGWQGMVDGWYGYHHSNEQGSGYAADKESTQKAIDGVTNKVNSIIDKMNTQFEAVGREFNNLERRI
ENLNKKMEDGFLDVWTYNAELLVLMENERTLDFHDSNVKNLYDKVRLQLRDNAKELGNGCFEFYHKC
DNECMESVRNGTYDYPQYSEEARLKREEISGVGSGYIPEAPRDGQAYVRKDGEWVLLSTFLGSGLNDI
FEAQKIEWHEGHHHHHH*

>H5-FMLMI-foldon
MENIVLLLAIVSLVKSDQICIGYHANNSTEQVDTIMEKNVTVTHAQDILEKTHNGKLCDLNGVKPLILKDC
SVAGWLLGNPMCDEFIRVPEWSYIVERANPANDLCFPGSLNDYEELKHMLSRINHFEKIQIIPKSSWPN
HETSLGVSAACPYQGAPSFFRNVVWLIKKNDAYPTIKISYNNTNREDLLILWGIHHSNNAEEQTNLYKNPI
TYISVGTSTLNQRLAPKIATRSQVNGQRGRMDFFWTILKPDDAIHFESNGNFIAPEYAYKIVKKGDSTIMK
SGVEYGHCNTKCQTPVGAINSSMPFHNIHPLTIGECPKYVKSNKLVLATGLRNSPLRESRGLFGAIAGFI
EGGWQGMVDGWYGYHFSNEQGSGYAADKESTQKAIDGVTNMVNSIIDKMNTQFEAVGMEFNNLERR
IENLNKKMEDGFIDVWTYNAELLVLMLNERTLDFHDSNVKNLYDKVRLQLRDNAKELGNGCFEFYHKC

bioRxiv preprint doi: https://doi.org/10.1101/2025.07.30.667762; this version posted August 2, 2025. The copyright holder for this preprint (which was not certified by peer review) is the author/funder, who has granted bioRxiv a license to display the preprint in perpetuity. It is made available under aCC-BY 4.0 International license.

DNECMESVRNGTYDYPQYSEEARLKREEISGVGSGYIPEAPRDGQAYVRKDGEWVLLSTFLGSGLNDI
FEAQKIEWHEGHHHHHH*

>H5-RC_I_1
MENIVLLLAIVSLVKSDQICIGYHANNSTEQVDTIMEKNVTVTHAQDILEKTHNGKLCDLNGVKPLILKDC
SVAGWLLGNPMCDEFIRVPEWSYIVERANPANDLCFPGSLNDYEELKHMLSRINHFEKIQIIPKSSWPN
HETSLGVSAACPYQGAPSFFRNVVWLIKKNDAYPTIKISYNNTNREDLLILWGIHHSNNAEEQTNLYKNPI
TYISVGTSTLNQRLAPKIATRSQVNGQRGRMDFFWTILKPDDAIHFESNGNFIAPEYAYKIVKKGDSTIMK
SGVEYGHCNTKCQTPVGAINSSMPFHNIHPLTIGECPKYVKSNKLVLATGLRNSPLRESRGLFGAIAGFI
EGGWQGMVDGWYGYHHSNEQGSGYAADKESTQKAIDGVTNKVNSIIDKMNTQFEAVGREFNNLERRI
ENLNKKMEDGFLDVWTYNAELLVLMENERTLDFHDSNVKNLYDKVRLQLRDNAKELGNGCFEFYHKC
DNECMESVRNGTYDYPQYSEEARLKREEISGVGSGSGSGSGSGSPDEDLKAELAATEAIWLLRQGRP
EEVWKLMQRLYEKGDPALWAVLRALLRSGDEIAILIAWNFMQRIGSWGSLEHHHHHH*

>H5-FMLMI-RC_I_1
MENIVLLLAIVSLVKSDQICIGYHANNSTEQVDTIMEKNVTVTHAQDILEKTHNGKLCDLNGVKPLILKDC
SVAGWLLGNPMCDEFIRVPEWSYIVERANPANDLCFPGSLNDYEELKHMLSRINHFEKIQIIPKSSWPN
HETSLGVSAACPYQGAPSFFRNVVWLIKKNDAYPTIKISYNNTNREDLLILWGIHHSNNAEEQTNLYKNPI
TYISVGTSTLNQRLAPKIATRSQVNGQRGRMDFFWTILKPDDAIHFESNGNFIAPEYAYKIVKKGDSTIMK
SGVEYGHCNTKCQTPVGAINSSMPFHNIHPLTIGECPKYVKSNKLVLATGLRNSPLRESRGLFGAIAGFI
EGGWQGMVDGWYGYHFSNEQGSGYAADKESTQKAIDGVTNMVNSIIDKMNTQFEAVGMEFNNLERR
IENLNKKMEDGFIDVWTYNAELLVLMLNERTLDFHDSNVKNLYDKVRLQLRDNAKELGNGCFEFYHKC
DNECMESVRNGTYDYPQYSEEARLKREEISGVGSGSGSGSGSGSPDEDLKAELAATEAIWLLRQGRP
EEVWKLMQRLYEKGDPALWAVLRALLRSGDEIAILIAWNFMQRIGSWGSLEHHHHHH*

mRNA immunogens

>H5-foldon
MENIVLLLAIVSLVKSDQICIGYHANNSTEQVDTIMEKNVTVTHAQDILEKTHNGKLCDLNGVKPLILKDC
SVAGWLLGNPMCDEFIRVPEWSYIVERANPANDLCFPGSLNDYEELKHMLSRINHFEKIQIIPKSSWPN
HETSLGVSAACPYQGAPSFFRNVVWLIKKNDAYPTIKISYNNTNREDLLILWGIHHSNNAEEQTNLYKNPI
TYISVGTSTLNQRLAPKIATRSQVNGQRGRMDFFWTILKPDDAIHFESNGNFIAPEYAYKIVKKGDSTIMK
SGVEYGHCNTKCQTPVGAINSSMPFHNIHPLTIGECPKYVKSNKLVLATGLRNSPLRESRGLFGAIAGFI
EGGWQGMVDGWYGYHHSNEQGSGYAADKESTQKAIDGVTNKVNSIIDKMNTQFEAVGREFNNLERRI
ENLNKKMEDGFLDVWTYNAELLVLMENERTLDFHDSNVKNLYDKVRLQLRDNAKELGNGCFEFYHKC
DNECMESVRNGTYDYPQYSEEARLKREEISGVGSGYIPEAPRDGQAYVRKDGEWVLLSTFLGSGLNDI
FEAQKIEWHE

>H5-FMLMI-foldon
MENIVLLLAIVSLVKSDQICIGYHANNSTEQVDTIMEKNVTVTHAQDILEKTHNGKLCDLNGVKPLILKDC
SVAGWLLGNPMCDEFIRVPEWSYIVERANPANDLCFPGSLNDYEELKHMLSRINHFEKIQIIPKSSWPN
HETSLGVSAACPYQGAPSFFRNVVWLIKKNDAYPTIKISYNNTNREDLLILWGIHHSNNAEEQTNLYKNPI
TYISVGTSTLNQRLAPKIATRSQVNGQRGRMDFFWTILKPDDAIHFESNGNFIAPEYAYKIVKKGDSTIMK
SGVEYGHCNTKCQTPVGAINSSMPFHNIHPLTIGECPKYVKSNKLVLATGLRNSPLRESRGLFGAIAGFI

bioRxiv preprint doi: https://doi.org/10.1101/2025.07.30.667762; this version posted August 2, 2025. The copyright holder for this preprint (which was not certified by peer review) is the author/funder, who has granted bioRxiv a license to display the preprint in perpetuity. It is made available under aCC-BY 4.0 International license.

EGGWQGMVDGWYGYHFSNEQGSGYAADKESTQKAIDGVTNMVNSIIDKMNTQFEAVGMEFNNLERR
IENLNKKMEDGFIDVWTYNAELLVLMLNERTLDFHDSNVKNLYDKVRLQLRDNAKELGNGCFEFYHKC
DNECMESVRNGTYDYPQYSEEARLKREEISGVGSGYIPEAPRDGQAYVRKDGEWVLLSTFLGSGLNDI
FEAQKIEWHE

>H5-RC_I_1
MENIVLLLAIVSLVKSDQICIGYHANNSTEQVDTIMEKNVTVTHAQDILEKTHNGKLCDLNGVKPLILKDC
SVAGWLLGNPMCDEFIRVPEWSYIVERANPANDLCFPGSLNDYEELKHMLSRINHFEKIQIIPKSSWPN
HETSLGVSAACPYQGAPSFFRNVVWLIKKNDAYPTIKISYNNTNREDLLILWGIHHSNNAEEQTNLYKNPI
TYISVGTSTLNQRLAPKIATRSQVNGQRGRMDFFWTILKPDDAIHFESNGNFIAPEYAYKIVKKGDSTIMK
SGVEYGHCNTKCQTPVGAINSSMPFHNIHPLTIGECPKYVKSNKLVLATGLRNSPLRESRGLFGAIAGFI
EGGWQGMVDGWYGYHHSNEQGSGYAADKESTQKAIDGVTNKVNSIIDKMNTQFEAVGREFNNLERRI
ENLNKKMEDGFLDVWTYNAELLVLMENERTLDFHDSNVKNLYDKVRLQLRDNAKELGNGCFEFYHKC
DNECMESVRNGTYDYPQYSEEARLKREEISGVGSGSGSGSGSGSPDEDLKAELAATEAIWLLRQGRP
EEVWKLMQRLYEKGDPALWAVLRALLRSGDEIAILIAWNFMQRIGSWGSLE

>H5-FMLMI-RC_I_1
MENIVLLLAIVSLVKSDQICIGYHANNSTEQVDTIMEKNVTVTHAQDILEKTHNGKLCDLNGVKPLILKDC
SVAGWLLGNPMCDEFIRVPEWSYIVERANPANDLCFPGSLNDYEELKHMLSRINHFEKIQIIPKSSWPN
HETSLGVSAACPYQGAPSFFRNVVWLIKKNDAYPTIKISYNNTNREDLLILWGIHHSNNAEEQTNLYKNPI
TYISVGTSTLNQRLAPKIATRSQVNGQRGRMDFFWTILKPDDAIHFESNGNFIAPEYAYKIVKKGDSTIMK
SGVEYGHCNTKCQTPVGAINSSMPFHNIHPLTIGECPKYVKSNKLVLATGLRNSPLRESRGLFGAIAGFI
EGGWQGMVDGWYGYHFSNEQGSGYAADKESTQKAIDGVTNMVNSIIDKMNTQFEAVGMEFNNLERR
IENLNKKMEDGFIDVWTYNAELLVLMLNERTLDFHDSNVKNLYDKVRLQLRDNAKELGNGCFEFYHKC
DNECMESVRNGTYDYPQYSEEARLKREEISGVGSGSGSGSGSGSPDEDLKAELAATEAIWLLRQGRP
EEVWKLMQRLYEKGDPALWAVLRALLRSGDEIAILIAWNFMQRIGSWGSLE

>H5-membrane-anchored
MENIVLLLAIVSLVKSDQICIGYHANNSTEQVDTIMEKNVTVTHAQDILEKTHNGKLCDLNGVKPLILKDC
SVAGWLLGNPMCDEFIRVPEWSYIVERANPANDLCFPGSLNDYEELKHMLSRINHFEKIQIIPKSSWPN
HETSLGVSAACPYQGAPSFFRNVVWLIKKNDAYPTIKISYNNTNREDLLILWGIHHSNNAEEQTNLYKNPI
TYISVGTSTLNQRLAPKIATRSQVNGQRGRMDFFWTILKPDDAIHFESNGNFIAPEYAYKIVKKGDSTIMK
SGVEYGHCNTKCQTPVGAINSSMPFHNIHPLTIGECPKYVKSNKLVLATGLRNSPLRESRGLFGAIAGFI
EGGWQGMVDGWYGYHHSNEQGSGYAADKESTQKAIDGVTNKVNSIIDKMNTQFEAVGREFNNLERRI
ENLNKKMEDGFLDVWTYNAELLVLMENERTLDFHDSNVKNLYDKVRLQLRDNAKELGNGCFEFYHKC
DNECMESVRNGTYDYPQYSEEARLKREEISGVKLESVGTYQILSIYSTAASSLALAIMMAGLSLWMCSN
GSLQCRICI

>H5-FMLMI-membrane-anchored
MENIVLLLAIVSLVKSDQICIGYHANNSTEQVDTIMEKNVTVTHAQDILEKTHNGKLCDLNGVKPLILKDC
SVAGWLLGNPMCDEFIRVPEWSYIVERANPANDLCFPGSLNDYEELKHMLSRINHFEKIQIIPKSSWPN
HETSLGVSAACPYQGAPSFFRNVVWLIKKNDAYPTIKISYNNTNREDLLILWGIHHSNNAEEQTNLYKNPI
TYISVGTSTLNQRLAPKIATRSQVNGQRGRMDFFWTILKPDDAIHFESNGNFIAPEYAYKIVKKGDSTIMK
SGVEYGHCNTKCQTPVGAINSSMPFHNIHPLTIGECPKYVKSNKLVLATGLRNSPLRESRGLFGAIAGFI

bioRxiv preprint doi: https://doi.org/10.1101/2025.07.30.667762; this version posted August 2, 2025. The copyright holder for this preprint (which was not certified by peer review) is the author/funder, who has granted bioRxiv a license to display the preprint in perpetuity. It is made available under aCC-BY 4.0 International license.

EGGWQGMVDGWYGYHFSNEQGSGYAADKESTQKAIDGVTNMVNSIIDKMNTQFEAVGMEFNNLERR
IENLNKKMEDGFIDVWTYNAELLVLMLNERTLDFHDSNVKNLYDKVRLQLRDNAKELGNGCFEFYHKC
DNECMESVRNGTYDYPQYSEEARLKREEISGVKLESVGTYQILSIYSTAASSLALAIMMAGLSLWMCSN
GSLQCRICI

ELISA+ns-EMPEM antigen

>H5-I53_dn5B
MENIVLLLAIVSLVKSDQICIGYHANNSTEQVDTIMEKNVTVTHAQDILEKTHNGKLCDLNGVKPLILKDC
SVAGWLLGNPMCDEFIRVPEWSYIVERANPANDLCFPGSLNDYEELKHMLSRINHFEKIQIIPKSSWPN
HETSLGVSAACPYQGAPSFFRNVVWLIKKNDAYPTIKISYNNTNREDLLILWGIHHSNNAEEQTNLYKNPI
TYISVGTSTLNQRLAPKIATRSQVNGQRGRMDFFWTILKPDDAIHFESNGNFIAPEYAYKIVKKGDSTIMK
SGVEYGHCNTKCQTPVGAINSSMPFHNIHPLTIGECPKYVKSNKLVLATGLRNSPLRESRGLFGAIAGFI
EGGWQGMVDGWYGYHHSNEQGSGYAADKESTQKAIDGVTNKVNSIIDKMNTQFEAVGREFNNLERRI
ENLNKKMEDGFLDVWTYNAELLVLMENERTLDFHDSNVKNLYDKVRLQLRDNAKELGNGCFEFYHKC
DNECMESVRNGTYDYPQYSEEARLKREEISGVSAEEAELAYLLGELAYKLGEYRIAIRAYRIALKRDPNN
AEAWYNLGNAYYKQGRYREAIEYYQKALELDPNNAEAWYNLGNAYYERGEYEEAIEYYRKALRLDPNN
ADAMQNLLNAKMREEGGWELQHHHHHH*

Table S2. CryoEM data collection and refinement statistics.

	H5-FMLMI-foldon<br>PDB 9PR3<br>EMD 71796
**Data collection and processing**	
Magnification (×)	105,000
Voltage (kV)	300
Electron exposure (e^{-}/Å^2)	60
Defocus range (μm)	-0.5 - -2
Pixel size (Å)	0.843
Symmetry imposed	C3
Final particle images (no.)	54,035
Map resolution (Å)	3.2
FSC threshold	0.143
Map sharpening *B* factor (Å^2)	-84
**Validation**	
MolProbity score	1.96

bioRxiv preprint doi: https://doi.org/10.1101/2025.07.30.667762; this version posted August 2, 2025. The copyright holder for this preprint (which was not certified by peer review) is the author/funder, who has granted bioRxiv a license to display the preprint in perpetuity. It is made available under aCC-BY 4.0 International license.

Clashscore	8.44
Poor rotamers (%)	0.5
Ramachandran plot	
Favored (%)	91.58
Allowed (%)	8.32
Disallowed (%)	0.1
